# Antibacterial action of slightly acidic electrolytic water against *Cronobacter sakazakii* and its application as a disinfectant on high-risk contact surfaces

**DOI:** 10.3389/fmicb.2024.1314362

**Published:** 2024-01-31

**Authors:** Ling Guo, Jing Han, Yanyan Wang, Yajing Chang, Wenxuan Qu, Chaoxin Man, Peng Fei, Yujun Jiang

**Affiliations:** ^1^Key Laboratory of Dairy Science, Ministry of Education, College of Food Science, Northeast Agricultural University, Harbin, China; ^2^Food Laboratory of Zhongyuan, Luohe, China; ^3^School of Zhang Zhongjing Health Care and Food, Nanyang Institute of Technology, Nanyang, China

**Keywords:** slightly acidic electrolytic water, *Cronobacter sakazakii*, antibacterial mechanism, application, powdered infant formula

## Abstract

Powdered infant formula (PIF) is prone to *Cronobacter sakazakii* (*C. sakazakii*) contamination, which can result in infections that endanger the lives of newborns and infants. Slightly acidic electrolytic water (SAEW) has shown antibacterial effects on a variety of foodborne pathogens and has a wide applicability in the food industry. Here, the antibacterial activity of SAEW against *C. sakazakii* and its use as a disinfectant on contact surfaces with high infection transmission risk were investigated. The inactivation of SAEW on *C. sakazakii* was positively correlated to the SAEW concentration and treatment time. The antibacterial effect of SAEW was achieved by decreasing the intracellular adenosine triphosphate (ATP), K^+^, protein, and DNA contents of *C. sakazakii*, reducing the intracellular pH (pH_in_) and destroying the cell morphology, which led to inactivation of *C. sakazakii* ultimately. To test the applicability of this study, the results showed that approximately 10^3^ CFU/cm^2^ of *C. sakazakii* were successfully inactivated on stainless steel and rubber surfaces after a 30 mg/L SAEW treatment for 20 s. These results indicate the antibacterial mechanism and potential application of SAEW against *C. sakazakii*, as well as a new strategy for the prevention and control of *C. sakazakii* on stainless steel and rubber surfaces.

## 1 Introduction

*Cronobacter sakazakii* (*C. sakazakii*) is a Gram-negative and non-spore-forming bacterium classified to the *Enterobacteriaceae*, which is an opportunistic foodborne pathogen. Neonatal meningitis, septicemia, and necrotizing enterocolitis are common illnesses of *C. sakazakii* infections. The death rate from *C. sakazakii* infections varies from 40 to 80% in the absence of effective treatment (Yan and Fanning, 2015; Phair et al., 2022).

Food contamination caused by *C. sakazakii* has received increasing attention because of its high osmotic pressure tolerance, good survival ability in low-moisture environments, and subsequent cross-contamination. Several foods and places have been reported to contain *C. sakazakii* contamination, powdered infant formula (PIF) in particular (Vojkovska et al., 2016; Lou et al., 2019). Gloves and stainless steel surfaces are the main external sources of food contamination by *C. sakazakii* (Lindsay et al., 2019). Therefore, the effective inhibition of *C. sakazakii* on the contact surfaces of dairy processing plants is very crucial. As of now, the food business has used peroxyacetic acid, organic acids, hydrogen peroxide, chlorine dioxide, and hydrogen peroxide as disinfectants (Sethi et al., 2020). Despite the fact that these disinfectants are effective, the food industry does not prioritize using them due to chemical management and environmental issues (Pacheappan et al., 2022).

As a new type of environmentally friendly antibacterial agent, slightly acidic electrolytic water (SAEW) has been widely used in food industry, agriculture and medical field (Roy et al., 2021). SAEW has the advantages of being less corrosive to equipment, less irritating to hands, and minimizing human health and safety issues in chemical disinfection (Zang et al., 2019). Electrolyzing diluted sodium chloride or hydrochloric acid solution is generally used as the raw material, from which SAEW can be generated using a special electrolytic device. By adjusting the devices, the pH value of SAEW can range from 5.0 to 6.5, and its oxidation–reduction potential (ORP) can be set to above 850 mV (Ye et al., 2017). The antimicrobial effect of SAEW is due to the interaction of its pH condition, redox potential and active chlorine (Li et al., 2023). SAEW can change the ultrastructure and permeability of *Staphylococcus aureus* and *E. coli*, leading to cell content leakage and inactivation (Ding et al., 2016; Kim et al., 2019). SAEW has the ability to effectively inhibit the growth of *Listeria innocua* on stainless steel tableware, rubber, and glass by limiting biofilm formation (Jeon et al., 2018). Spraying SAEW also can effectively reduce the number of *Staphylococcus epidermidis* in the air (Naka et al., 2020).

Although these investigations showed SAEW to have good antibacterial action against foodborne pathogens on surfaces that could potentially come into contact with food, few studies have reported the mechanism of action of SAEW against *C. sakazakii*. We hypothesize that SAEW can destroy the cell membrane of *C. sakazakii*, causing leakage of its intracellular contents and thus restraining the growth of *C. sakazakii.* As such, this work investigated the antibacterial effects of SAEW against *C. sakazakii* and evaluated the application of SAEW on rubber gloves and stainless steel surfaces. Our main objective is to offer a theoretical foundation for better SAEW use in the areas of food safety control, quality maintenance, and shelf-life extension.

## 2 Materials and methods

### 2.1 Activation and preservation of strains

The cryopreserved tube of *C. sakazakii* (ATCC 29544, American Type Culture Collection) stored at −80°C was removed and thawed. Then inoculated the *C. sakazakii* into sterile Luria-Bertani (LB) broth medium at 3% inoculum, cultured at 37°C for 8–12 h, and streaked on Trypticase Soy Agar (TSA) medium. The single colonies were inoculated into sterilized LB broth medium and incubated at 37°C for 24 h until the population reached 10^8^ CFU/mL. This bacterial suspension was used in relevant tests. In addition, the bacterial solution and glycerin were transferred in a 3:1 ratio into a 1.5 mL cryo-storage tube, which was then stored at −80°C.

### 2.2 Preparation of SAEW

The preparation of SAEW followed the method of Hao et al. (2017). Sodium chloride solution of 0.1% and hydrochloric acid solution of 0.1% were added to the SAEW generator (KH SAEW-1500, Kanghui Water Treatment Equipment Co., Ltd., Shandong, China) to obtain SAEW. The pH and oxidation–reduction potential (ORP) were measured by a pH/ORP meter (Model ST2100, Ohaosi Instruments Changzhou Limited, China), and the available chlorine concentration (ACC) was determined using the iodometric method (Zeng et al., 2010). SAEW was replaced with deionized water in blank control test. Table 1 shows the pH, ORP, and ACC of the SAEW in this study.

**Table 1:** Physicochemical properties of the SAEW.

Physicochemical property	Different concentrations of SAEW				
	10 mg/L	15 mg/L	20 mg/L	25 mg/L	30 mg/L
pH	6.13 ± 0.04^a^	5.94 ± 0.02^b^	5.92 ± 0.03^b^	5.82 ± 0.02^c^	5.78 ± 0.02^c^
ORP (mv)	840.67 ± 2.08^a^	872.00 ± 3.61^b^	902.33 ± 1.53^c^	916.00 ± 2.64^d^	924.33 ± 0.57^e^
ACC (mg/L)	10.34 ± 1.05^a^	14.83 ± 0.79^b^	20.02 ± 1.07^c^	25.68 ± 2.10^d^	30.95 ± 0.94^e^

Different uppercase (or lowercase) letters after the data in the same row (or column) indicate that one-way ANOVA of the mean values differs significantly (*p* < 0.05).

### 2.3 Determination of the antibacterial efficacy of SAEW

The 2 mL of *C. sakazakii* suspension (10^8^ CFU/mL) was treated with 4 mL of SAEW at different concentrations (0, 10, 15, 20, 25, and 30 mg/L) for 20 s, 40 s, and 60 s, respectively. According to Tao’s study, 1 mL of the mixture was transferred into a sterile tube containing 4 mL of neutralization buffer solution (0.85% NaCl solution and 0.5% sodium thiosulfate) to terminate the reaction (Tao et al., 2022). After 10 min, dilute the mixture to 10^5^ CFU/mL with sterile normal saline. The 100 μL diluent was plated on TSA medium. The colonies were counted following a 37°C overnight incubation (DHP-9272, Yiheng Technology Co., Ltd., Shanghai).

### 2.4 Measurement of intracellular ATP concentrations

This experiment referred to the method of Sánchez et al. (2010), with minor modifications. The 2 mL of *C. sakazakii* suspension (10^8^ CFU/mL) was treated with 4 mL of SAEW of different concentrations (0, 10, 20, and 30 mg/L) for 20 s, 40 s, or 60 s. Sterile normal saline was used as a control. The intracellular ATP concentrations were measured using an ATP assay kit (Biyuntian Biotechnology Co., Ltd., China). The supernatant was prepared by centrifuged at 12,000 × *g* for 5 min, and then stored the supernatant in ice box for later use.

### 2.5 Measurement of intracellular K^+^ concentrations

The K^+^ leakage was measured according to the study of Ding et al. (2016). The 2 mL of *C. sakazakii* suspension was treated with 4 mL of SAEW of different concentrations for 20 s, 40 s, or 60 s. Sterile normal saline was used as a control. The mixture was centrifuged at 8,000 × *g* for 10 min before being filtered through 0.22 μm filters (Pilot Experimental Equipment Co., Ltd., China). The filtrate was then used to measure the content of K^+^ by atomic absorption spectrophotometry (iCE3500, Saimer Fischer Technology Co., Ltd., China).

### 2.6 Measurement of intracellular protein concentrations

The protein leakage of the *C. sakazakii* cells was measured according to the Hao’s previous report (Hao et al., 2017). The 2 mL of cell suspension was treated with 4 mL of SAEW of different concentrations for 20 s, 40 s, or 60 s. The control was sterile normal saline. The samples were centrifuged at 8,000 × *g* for 10 min, and 1 mL of the supernatant was taken and 5 mL of the Coomassie brilliant blue solution was used to stain it (Solarbio Biotechnology Co., LTD., China). After staining for 5 min, the mixture was analyzed for protein leakage by ultraviolet–visible spectrophotometry (Qingdao Lubo Weiye Environmental Protection Technology Co., Ltd., China) at 595 nm.

### 2.7 Measurement of DNA fragmentation

The effect of DNA fragmentation caused by the SAEW on *C. sakazakii* was measured using agarose gel electrophoresis (AGE), according to previous report (Liu et al., 2011; Guo et al., 2019). The 2 mL of cell suspension was treated with 4 mL of SAEW of different concentrations for 20 s, 40 s, or 60 s. Sterile normal saline was used as a control. The genomic DNA was extracted using the bacterial genomic DNA extraction kit (Tiangen Biotech Co., Ltd., Beijing, China). The DNA samples were then added to the prepared agarose gelsand gel wells and stained with a fluorescent dye—ethidium bromide (EB). Finally, the electrophoresis was performed at 100 V for 30 min, from which the image was obtained from a gel imaging system (Tianchi Yicheng Biotechnology Co., LTD., Beijing, China).

### 2.8 Measurement of intracellular pH (pH_in_)

The changes in pH_in_ were determined following a protocol described by Guo et al. (2020b). First, the *C. sakazakii* cell suspension was washed twice with a 50 mmol/L HEPES buffer solution before being mixed with a 3 μmol/L pH_in_ fluorescent probe at 37°C for 20 min. The suspension was then washed continuously with a 50 mmol/L K_3_PO_4_ solution and resuspended again, to which a 10 μmol/L glucose solution was added, and the resulting suspension was cultured at 37°C for 30 min. Finally, the sample was washed twice with a neutral PBS buffer solution, and the bacteria were suspended again, then stored on ice for later use.

The *C. sakazakii* suspension treated as described above was taken and treated with 4 mL of SAEW of different concentrations for 20 s, 40 s, or 60 s. In addition, sterile normal saline was used as a control. The completed reaction samples were added to a 96-well opaque black plate, and the fluorescence intensities of the buffer solutions at various pH_in_ values (3, 4, 5, 6, 7, 8, 9, 10) were measured using a microplate reader (Tecan Infinite 200 PRO, Putian Xinqiao Technology Co., Ltd., Beijing) at a wavelength of 520 nm for the emission and 490 nm for the excitation.

### 2.9 Transmission electron microscopy (TEM) analysis

The cellular morphology of *C. sakazakii* treated with different concentrations of SAEW (0, 15, and 30 mg/L) for 20 s, 40, or 60 s was observed by a transmission electron microscope (*/HT7800, Hitachi, Japan) following the previous report of Fei et al. (2019). The control was sterile normal saline. After treatments, the *C. sakazakii* cells were centrifuged at 8,000 × *g* for 10 min and then mixed with 2.5% glutaraldehyde at 4°C for 2 h. The cells were rinsed three times with 0.1 M phosphate buffer (pH = 7.2) for 15 min each before being fixed with 1% osmium tetraoxide for 120 min and rinsed three times more. The cells were treated with different concentrations of ethanol (50, 70, 90, 100%) and then treated with the mixture of 100% ethanol and 100% acetone. The samples were infiltrated with acetone and embedding solution, followed by embedding, sectioning and positive staining of the samples. Finally, observed under TEM.

### 2.10 Antibacterial activity of SAEW on stainless steel and rubber surfaces

The antibacterial activity of SAEW on stainless steel and rubber surfaces were analyzed following a previously reported protocol (Huang et al., 2006). The stainless steel and rubber had thicknesses of 0.05 mm and 0.5 mm (Harbin Hardware Market., China), respectively, and were cut into a sheet with dimensions of 25 (5 × 5) cm^2^. The squares were immersed in a *C. sakazakii* suspension (10^8^ CFU/mL) for 5 min after sterilization and dried in a laminar flow cabinet for 20 min at room temperature (Jiasheng Together Manufacturing Co., LTD., China). Then the sterile cotton swabs were used to wipe the squares, and the swabs were thoroughly washed with 5 mL of sterile sodium chloride solution. Then the sterile cotton swabs were used to wiped the squares and washed the swabs adequately with 5 mL of sterile sodium chloride solution. The solution was properly diluted, plated on the TSA medium, and incubated overnight at 37°C. Finally, colony counting was performed to obtain the initial colony number on the surfaces.

The SAEW of different concentrations were sprayed on the surface of the above prepared squares with the bacterial solution, and treated for 20 s, 40 s and 60 s, respectively. The bacteria adhering to the stainless steel sheet squares and rubber sheet squares were removed with sterile cotton swabs, which were then thoroughly washed with the sterile sodium chloride solution. The diluted broth was plated on the TSA solid medium, and incubated overnight in an incubator at 37°C for colony counting.

### 2.11 Statistical analysis

Experiments were repeated in triplicate and data were analyzed using SPSS 19.0 software (SPSS, Chicago, IL). All data are expressed as the mean values ± Standard deviation (SD). The statistical differences at 5% significance level among means were determined by one-way analysis of variance (ANOVA).

## 3 Results and discussion

### 3.1 The antibacterial activity of SAEW against *Cronobacter sakazakii*

The antibacterial effect of SAEW with different concentrations against *C. sakazakii* was shown in Table 2. After the treatment of different concentrations of SAEW, the number of *C. sakazakii* significantly decreased. Furthermore, when the reaction time increased from 20 to 60 s, the total number of *C. sakazakii* significantly decreased (*p* < 0.05). The SAEW at a concentration of 30 mg/L inactivated about 10^8^ CFU/mL of *C. sakazakii* strains within 60 s.

**Table 2:** The colony number of *C. sakazakii* ATCC 29544 with different concentration of SAEW.

The concentration of SAEW (mg/L)	Action time (s)		
	20	40	60
0	8.11 ± 0.05		
10	4.42 ± 0.47^Aa^	3.71 ± 0.04^Ba^	3.29 ± 0.03^Ba^
15	3.77 ± 0.57^Aa^	3.02 ± 0.04^Bb^	2.61 ± 0.06^Bb^
20	2.76 ± 0.05^Ab^	2.68 ± 0.05^Ac^	2.52 ± 0.08^Bc^
25	2.50 ± 0.03^Ab^	2.34 ± 0.02^Bd^	2.27 ± 0.06^Bd^
30	2.01 ± 0.16^Ab^	1.73 ± 0.15^Be^	0.00 ± 0.00^Ce^
35	0.67 ± 1.15^Ac^	0.00 ± 0.00^Af^	0.00 ± 0.00^Ae^
40	0.00 ± 0.00^Ac^	0.00 ± 0.00^Af^	0.00 ± 0.00^Ae^

Different uppercase (or lowercase) letters after the data in the same row (or column) indicate that one-way ANOVA of the mean values differs significantly (*p* < 0.05).

Previous research has found that SAEW can inhibit the growth of *Rhizopus stolonifer* colonies, and that the effect of inhibition increases with concentration (Li et al., 2021). The effect of acidic electrolyzed water can cause the decline of *C. sakazakii* colony on the surface of pear, melon and apple with values of 1.3, 1.7 and 1.8 log CFU/g, respectively, and there is no significant difference in the decline rate of each colony (Santo et al., 2016). In the present study, *C. sakazakii* significantly decreased with an increase in SAEW concentrations. In addition, due to its slightly acidic pH, SAEW does not corrode processing equipment or irritate producers’ hands as severely as strong acidic electrolyzed water (Abadias et al., 2008; Issa-Zacharia et al., 2010).

### 3.2 Changes in intracellular ATP content

**Figure 1:**
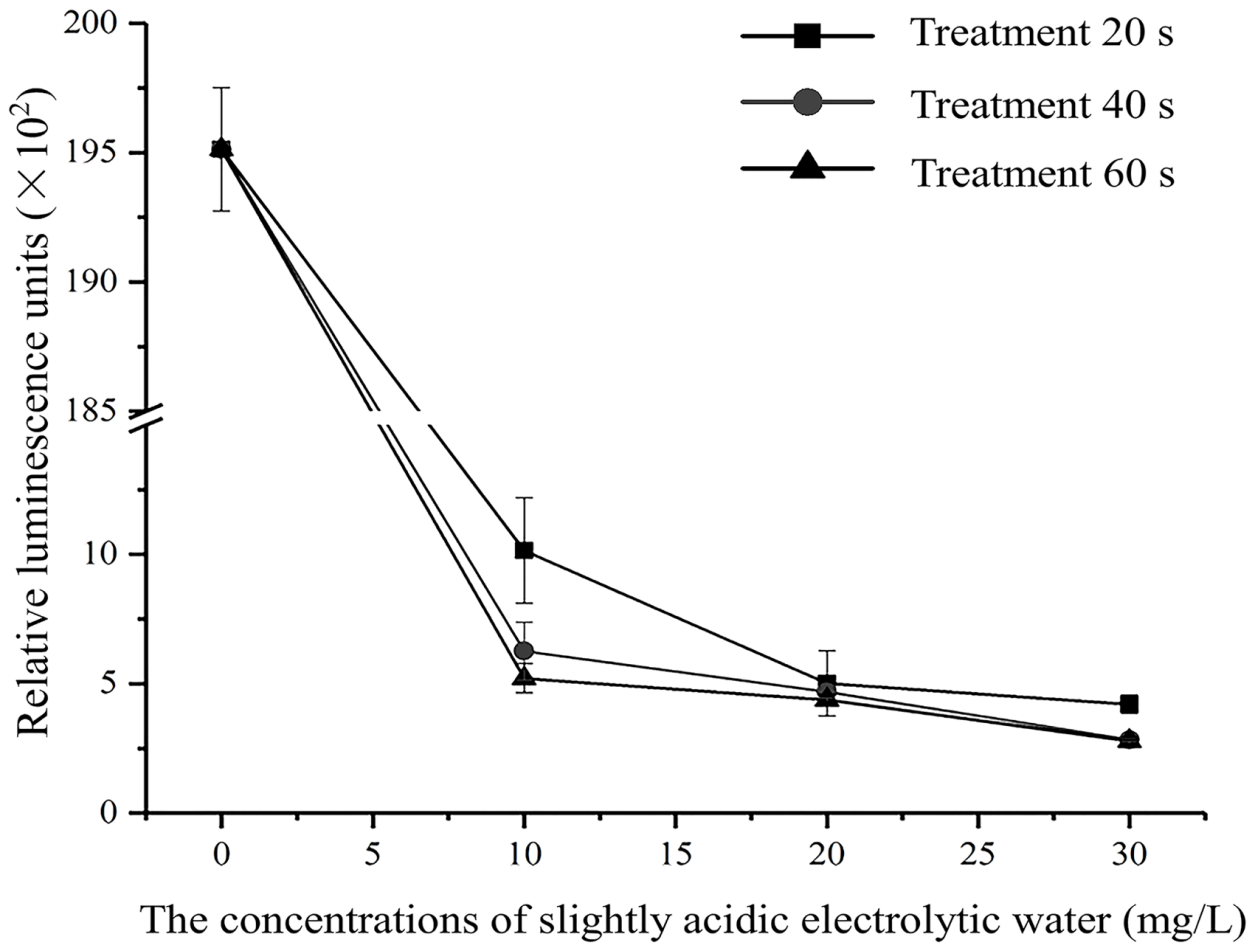
Effect of different concentration of SAEW in intracellular ATP content of *C. sakazakii* ATCC 29544.

The effects of different SAEW concentrations on the intracellular ATP content of *C. sakazakii* were determined, as shown in Figure 1. The SAEW concentration and time extension were found to be inversely related to the relative luminescence value, indicating that the intracellular ATP content of *C. sakazakii* showed a decreasing trend and that the content of intracellular ATP was influenced by both the SAEW concentration and action time. Similarly, the reduction of intracellular ATP also occurred during the SAEW against *Pseudomonas fluoresces*, *E. coli*, *S. aureus*, and *Bacillus subtilis* (Hao et al., 2017; Cai et al., 2019). In general, the intracellular ATP concentration of bacteria was stable but showed lower stability once the cell membrane was damaged (Kacprzyk et al., 2007). Therefore, it is presumed that the treatment of SAEW can damage the cell membrane of *C. sakazakii*, destroying the vitality of *C. sakazakii*.

### 3.3 Changes in the amount of K^+^ leakage

**Figure 2:**
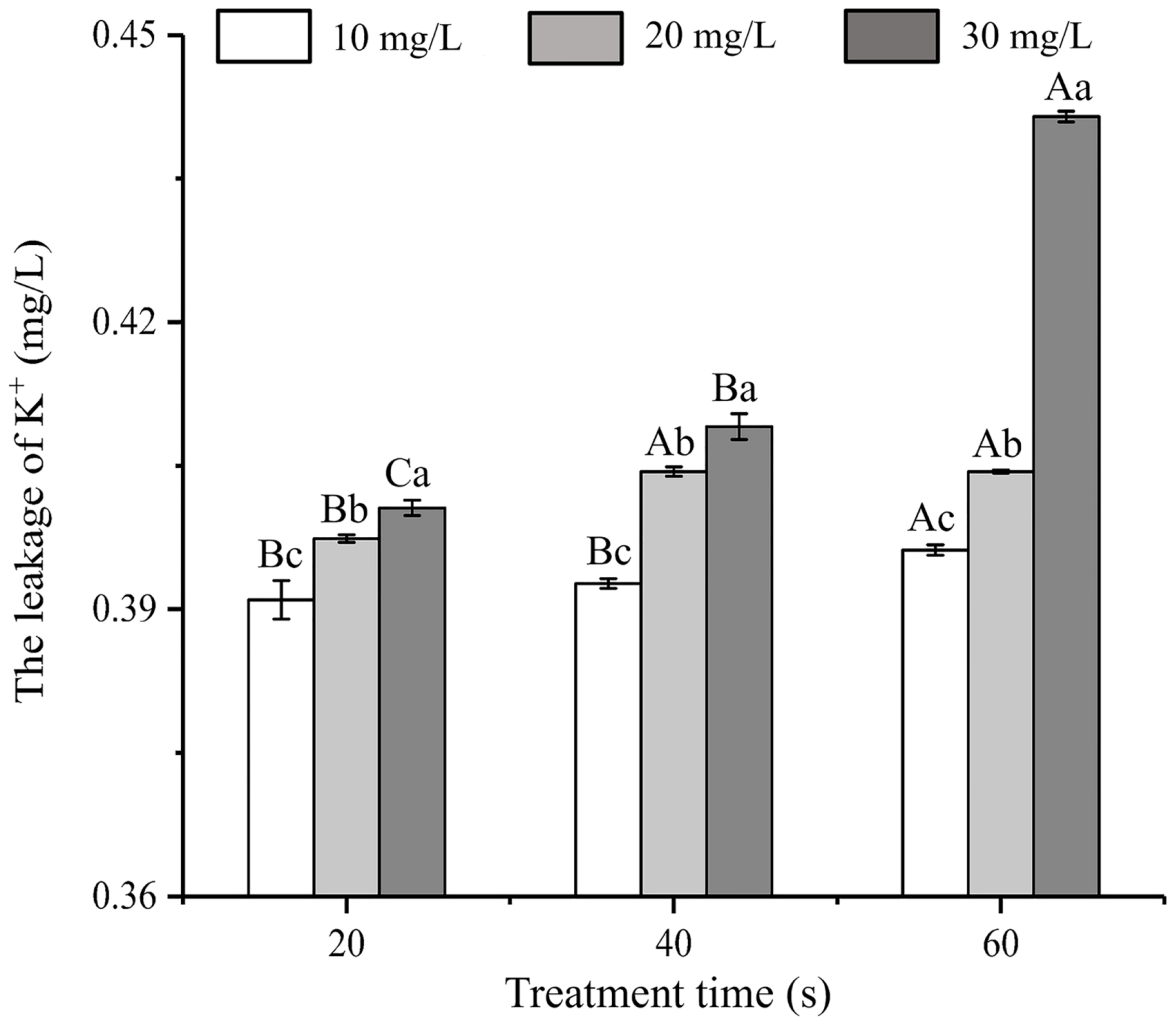
Effect of different concentration of SAEW in intracellular K^+^ content of *C. sakazakii* ATCC 29544.

As shown in Figure 2, the effects of SAEW on K^+^ leakage at different concentrations and treatment times were analyzed. When treated with SAEW for 60 s, the K^+^ leakage of *C. sakazakii* increased with the concentration of SAEW, and the difference of leakage was significant (*p* < 0.05). Previous researches have explored the inhibitory effect of SAEW on *B. subtilis* and *S. aureus*, from which K^+^ leakage exhibited a positive correlation with the concentration and action time of SAEW (Tang et al., 2011; Ding et al., 2016). Zeng et al. (2010) reported the effect of SAEW (30.20 mg/L) on K^+^ leakage in *S. aureus* and *E. coli*, which results showed that K^+^ leakage in cells rapidly increased at the beginning of the SAEW treatment (approximately 1 min), but tended to slow down at the later stages. The large loss of K^+^ may be due to SAEW-induced denaturation of the K^+^ channel transmembrane protein (Hebert et al., 2005). The K^+^ leakage led to an imbalance of the ion channels and seriously damaged the physiological function of the bacteria, thus promoting bacterial death (Tao et al., 2022).

### 3.4 Protein leakage

**Figure 3:**
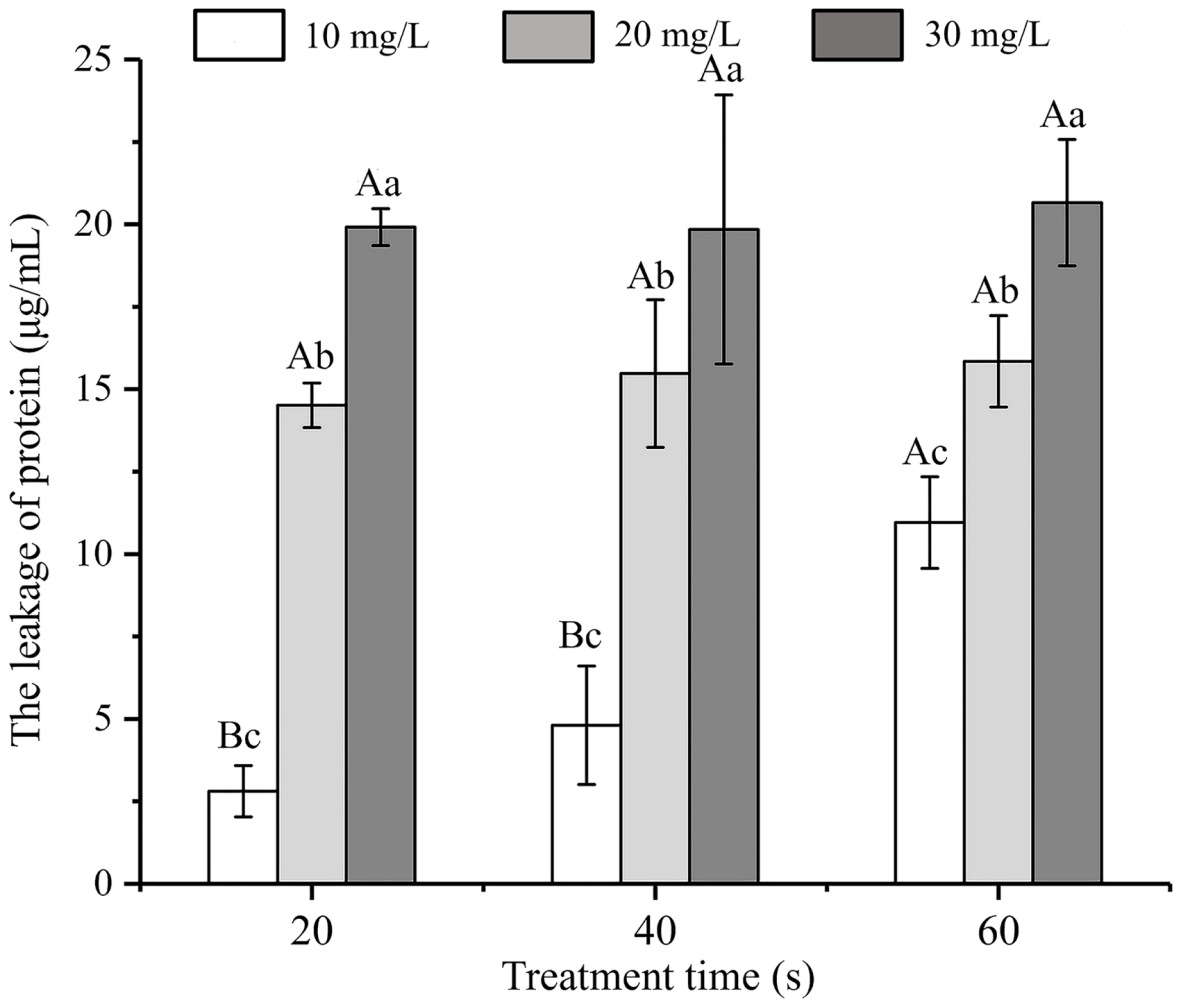
Effect of different concentration of SAEW in intracellular protein content of *C. sakazakii* ATCC 29544.

The influence of SAEW at different concentrations on protein leakage from *C. sakazakii* is shown in Figure 3. At the same treatment time, the protein leakage of *C. sakazakii* increased with the increase in SAEW concentration (*p* < 0.05). At identical concentration, the protein leakage showed a positive correlation with the action time of SAEW, indicating that the concentration of SAEW had a significant impact on the protein leakage of *C. sakazakii*. As the basic substance of bacterial life activities, excessive protein leakage can affect the normal metabolic function of *C. sakazakii,* leading to bacterial inactivation (Wang et al., 2015). Zeng also observed that SAEW caused protein leakage in *S. aureus* and *E. coli* (Zeng et al., 2010). Given that protein leakage is irreversible, higher protein leakage is expected at longer processing times. Therefore, SAEW can lead to the inactivation of *C. sakazakii* due to the leakage of bacterial protein.

### 3.5 DNA cleavage analysis

**Figure 4:**
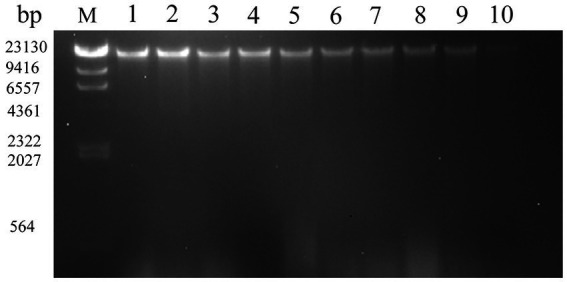
Effect of different concentration of SAEW in intracellular DNA content of *C. sakazakii* ATCC 29544. Lane M: marker; Lane 1: blank control; Lanes 2, 3, 4: the concentration of SAEW is 10 mg/L; Lanes 5, 6, 7: the concentration of SAEW is 20 mg/L; Lanes 8, 9, 10: the concentration of SAEW is 30 mg/L; Lanes 2, 5, 8: the action time of SAEW is 20 s; Lanes 3, 6, 9: the action time of SAEW is 40 s; Lanes 4, 7, 10: the action time of SAEW is 60 s.

The growth, development, and heredity of bacteria are inseparable from the control of DNA. DNA loss is very harmful to all cells (Cui et al., 2018). The effects of SAEW on the DNA of *C. sakazakii* are shown in Figure 4. The results showed a lighter and eventual disappearance of the strip color as the SAEW concentration increased. When the concentrations were the same, the color of the strips gradually became lighter as the action time increased, but the difference was not noticeable. The streaks of bacterial DNA were vague and imprecise at the SAEW concentration of 30 mg/L. The DNA bands of *C. sakazakii* disappeared after treatment with 30 mg/L SAEW for 60 s. The results demonstrated that the brightness of SAEW-treated bacterial DNA bands was lower than the control group, suggesting that DNA leakage occurred after the SAEW treatment in *C. sakazakii*. Other researchers used SAEW in *S. aureus*, *E. coli*, *B. subtilis*, and *Listeria monocytogenes*, from which SAEW reduced the intracellular DNA of these pathogens (Zeng et al., 2010; Hao et al., 2017). The decrease of DNA in bacterial cells, it is supposed that a result of SAEW damage toward bacterial cell membranes, resulting in macro-molecular nucleotide leakage, which combined with DNA in the groove mode and inhibited normal bacterial functions, ultimately leading to bacterial death (Wu et al., 2017).

### 3.6 Changes in intracellular pH (pH_in_)

**Figure 5:**
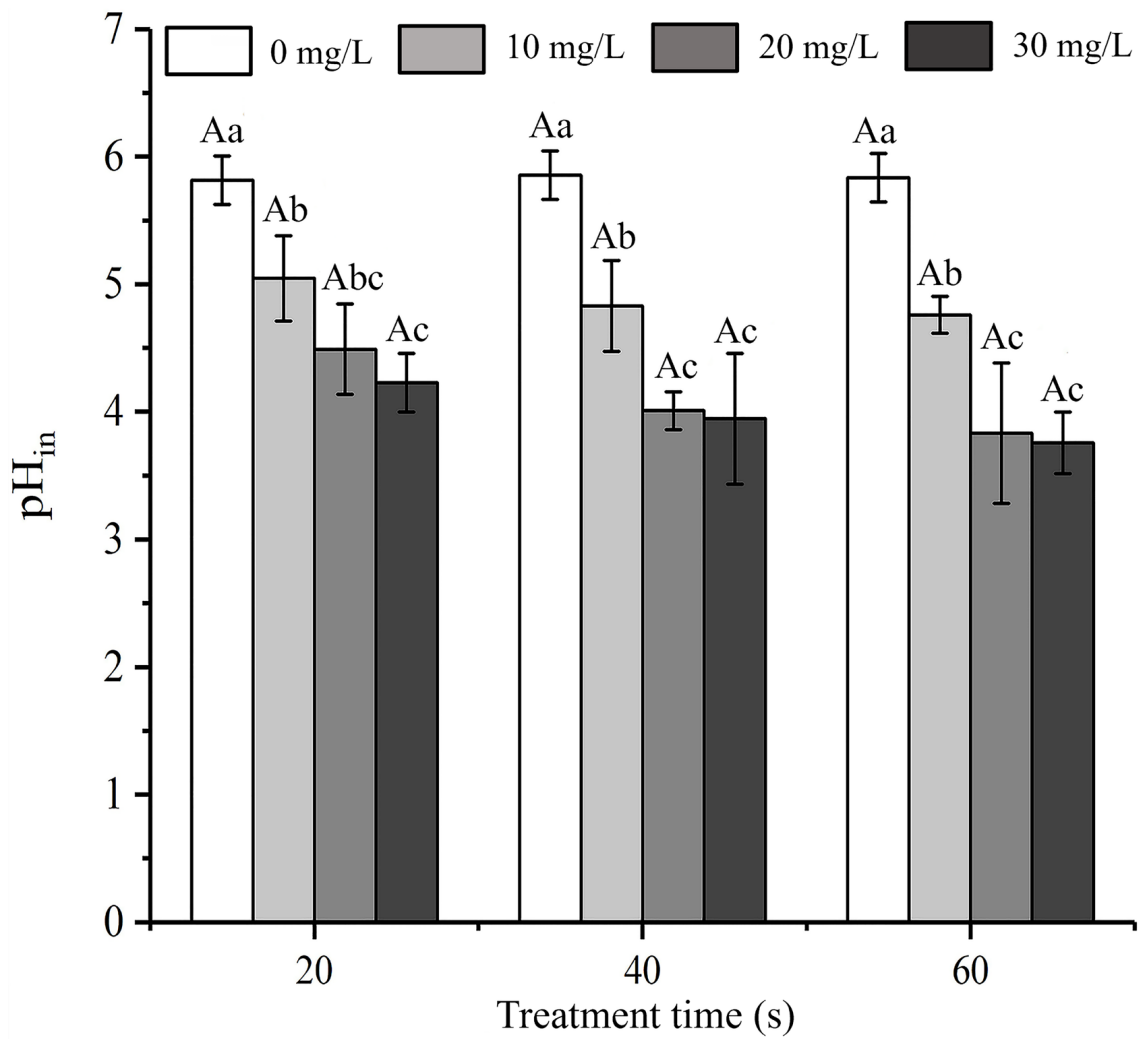
Effect of different concentration of SAEW on the intracellular pH of *C. sakazakii* ATCC 29544.

SAEW can change the pH_in_ gradient, which can increase the sensitivity of the cell membrane, thus promoting cell membrane destruction and cell inactivation (Hricova et al., 2008). The influence of SAEW on the pH_in_ of *C. sakazakii* is shown in Figure 5. The pH_in_ of bacteria decreased at longer action times at the same concentration (*p* < 0.05). The initial pH_in_ of *C. sakazakii* control group was approximately 5.97 ± 0.19. According to Figure 5, the pH_in_ values of *C. sakazakii* treated with SAEW were all lower than the normal range. The previous study also found that acidic electrolytic water could significantly lower the pH_in_ of *Pseudomonas fluorescens* (Cai et al., 2019), which is consistent with this study. It is assumed that SAEW provided a slightly acidic environment around the bacteria to lower the pH_in_ of *C. sakazakii*, thereby altering the permeability of the cell membrane and resulting in inactivation.

### 3.7 Changes in cell morphology

**Figure 6:**
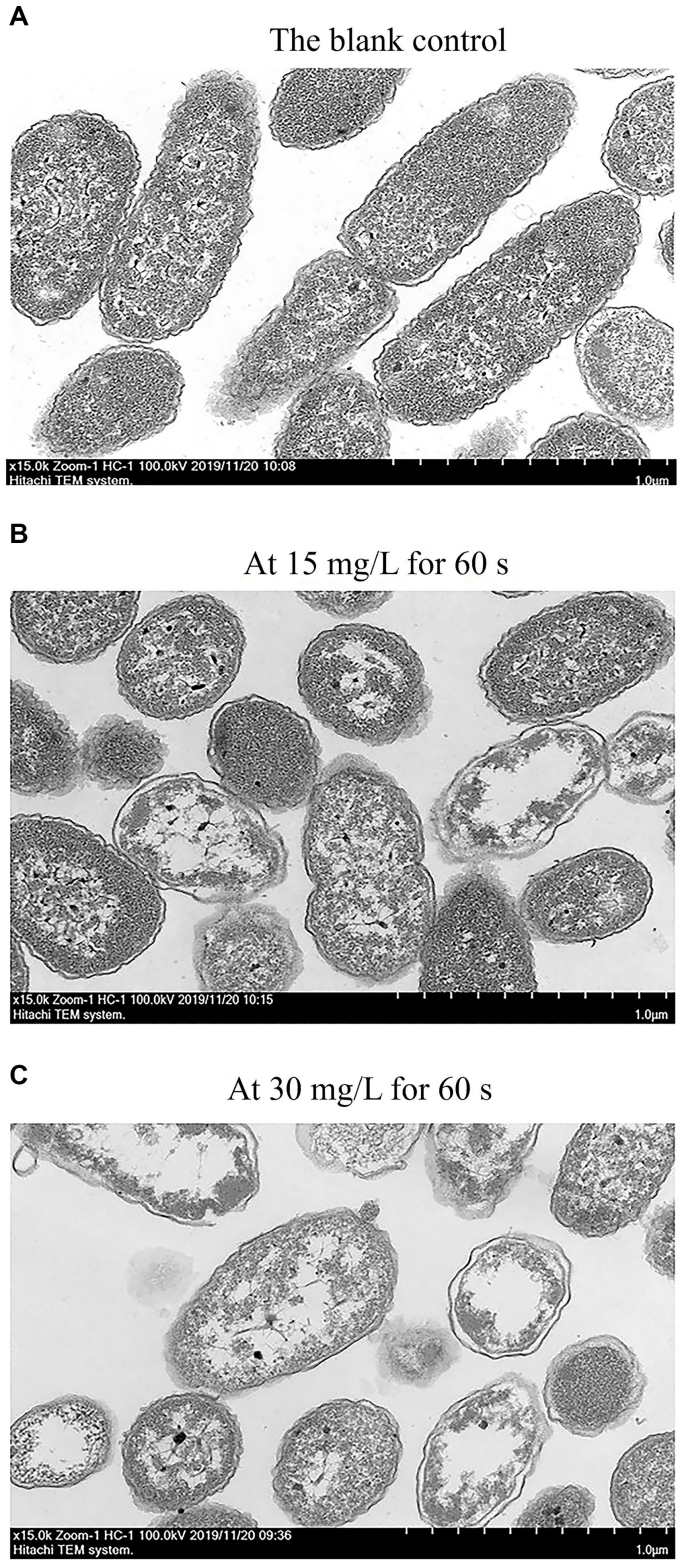
The TEM of *C. sakazakii* ATCC 29544 after treated with different concentration of SAEW. **(A)** The blank control. **(B)** At 15 mg/L for 60 s. **(C)** At 30 mg/L for 60 s.

Transmission electron microscopy (TEM) was used to visually observe any changes in cell morphology and cytoplasm (Guo et al., 2020a). Figure 6 presents the TEM diagram of *C. sakazakii* treated with SAEW at different concentrations. The TEM image of *C. sakazakii* after treatment with 15 mg/L SAEW for 60 s was shown in Figure 6B, which exhibited cell wall and cell membrane dissolution. As a result, the cell contents showed an uneven distribution and even leaked and migrated to the edge. Figure 6C presents the TEM image of *C. sakazakii* after the 30 mg/L SAEW treatment for 60 s, wherein most of *C. sakazakii* showed internal voids and serious plasmolysis. Overall, a large amount of cell contents leaked, and the cells showed serious damage. After being treated with SAEW, *C. sakazakii* exhibited changes in its cell morphology, membrane damage, and uneven cell content distribution and leakage. Moreover, the study have reported that the antibacterial effect of SAEW can also destroy the integrity of cell membranes of *Salmonella typhi*, *S. aureus*, and *Bacillus cereus*, thereby resulting in cell content leakage and thus affecting cell function (Kim et al., 2019).

### 3.8 Application of SAEW on the surfaces of stainless steel and rubber

**Figure 7:**
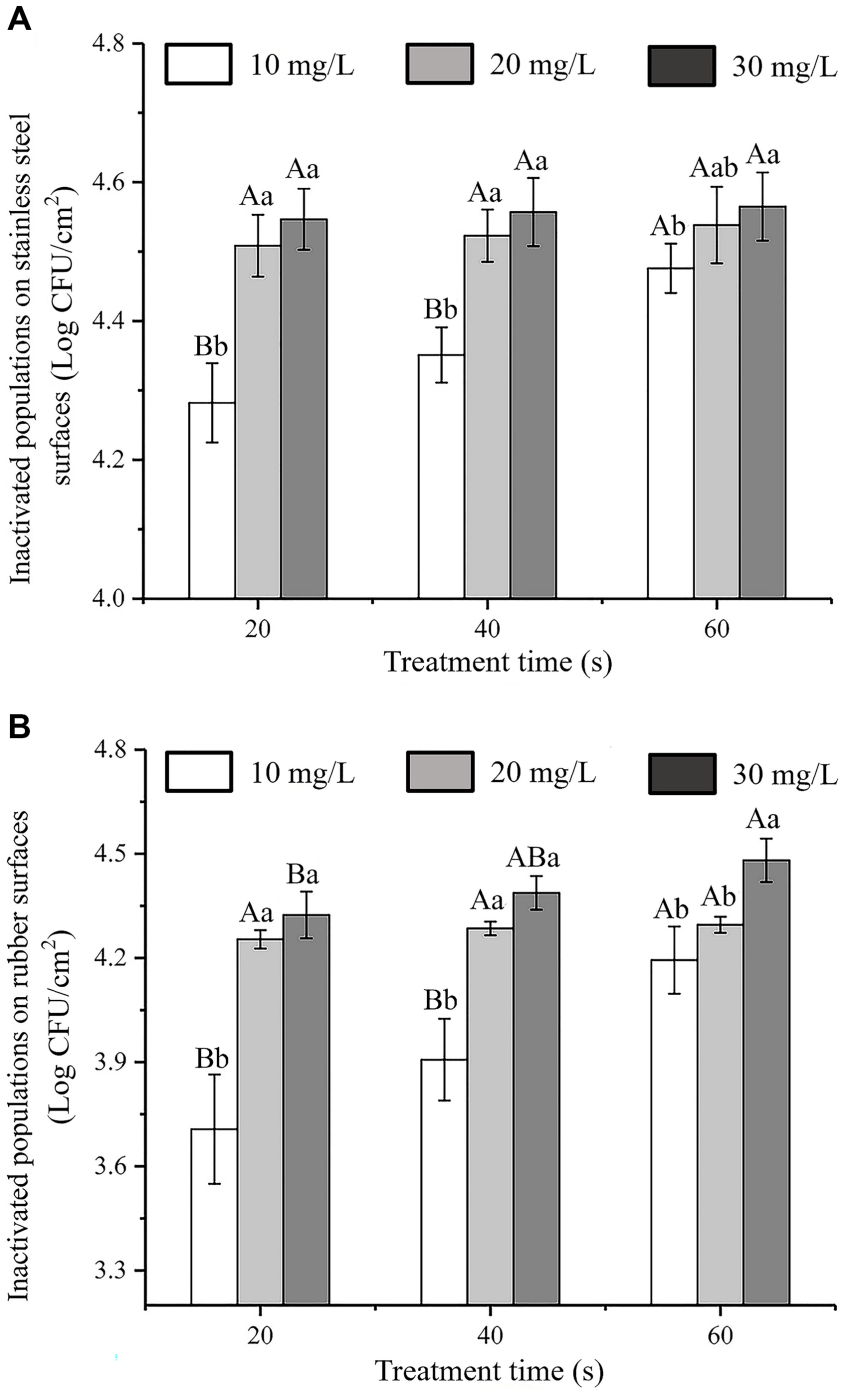
Antibacterial activity of SAEW against *C. sakazakii* ATCC 29544 inoculatedon to different surfaces. **(A)** Stainless steel surface. **(B)** Rubber surface.

The antibacterial effects of SAEW with different action times and concentrations against *C. sakazakii* inoculated on stainless steel and rubber surfaces are shown in Figures 7A,B, respectively. The initial inoculum amounts of *C. sakazakii* on the stainless steel and rubber surfaces were approximately 4.60 ± 0.05 log CFU/cm^2^ and 4.58 ± 0.06 log CFU/cm^2^, respectively. The number of *C. sakazakii* colonies inoculated on stainless steel and rubber decreased as SAEW concentration and action time increased. At a concentration of 30 mg/L and an action time of 60 s, almost all of the *C. sakazakii* colonies on the stainless steel and rubber surfaces were inactivated. The inactivation rates were 99.06 and 99.03%, respectively.

According to Chauhan’s study on the inhibition of *C. sakazakii* by organic acids, it was found that SAEW had better inhibition of *C. sakazakii* by comparison, and compared with Chang’s study, SAEW had better inhibition of bacteria on *C. sakazakii* than *Houttuynia cordata* Thunb. crude extract (Chauhan et al., 2022; Chang et al., 2023). In addition, the experimental results show that SAEW with low ACC and near neutral pH can sterilize *E. coli* and *Staphylococcus aureus* in a short period of time, and will become a potential alternative to NaOCl solutions commonly used in the food industry (Issa-Zacharia et al., 2010).

Previous research on the antibacterial activity of SAEW to against other pathogenic bacteria on food contact surfaces have reported that spraying with SAEW for 0.5–2.0 min resulted in a reduction of *E. coli*, *S. typhimurium*, and *S. aureus* on stainless steel by 3.44–6.01, 4.51–5.64, and 4.68–5.38 log CFU/cm^2^, respectively (Ni et al., 2016). Similarly, *L. monocytogenes* inactivation on natural rubber latex gloves was reported after a 5 min treatment with acid electrolytic water (Huang et al., 2006). The results of our study were consistent with those of earlier investigations, which demonstrated that SAEW had an effective inhibitory effect on *C. sakazakii* on the surfaces of stainless steel and rubber gloves.

In general, chemical disinfectants are used in the food industry to inhibit *C. sakazakii* on equipment and material surfaces (Chang et al., 2021). However, the prolonged and excessive use of chemical disinfectants can result in bacterial drug dependence or resistance, thus lowering their effectiveness, such that their potential impact cannot be ignored (Mc Carlie et al., 2020; Notcovich et al., 2020). Lindsay et al. (2019) showed that rubber gloves and stainless-steel facilities used by the staff were the main sources of *Cronobacter* species contamination in the dairy plants. Therefore, it is necessary to control the growth of *C. sakazakii* on stainless-steel facilities and rubber gloves.

## 4 Conclusion

This study demonstrated that SAEW has effective antibacterial activity against *C. sakazakii*. At an SAEW concentration of 30 mg/L, *C. sakazakii* was completely inactivated after 60 s of treatment. Higher antibacterial efficacy was also observed at increased times and concentrations. The mechanism of SAEW against *C. sakazakii* was illustrated by damage to the cell membrane as well as changes in cell membrane permeability and cell morphology, causing the cell contents leakage, including its intracellular ATP, K^+^, protein, and DNA. As a result, the ion balance inside and outside the cell was disrupted, thus decreasing the pH_in_ content, ultimately inactivating *C. sakazakii*. After treatment with 30 mg/L SAEW for 20 s, approximately 10^3^ CFU/cm^2^ of *C. sakazakii* on stainless steel and rubber surfaces were inactivated. Overall, this study suggests that SAEW is a potential convenient disinfectant for use in the dairy industry to inhibit *C. sakazakii* on stainless steel and rubber surfaces.

## Data availability statement

The original contributions presented in the study are included in the article/supplementary material, further inquiries can be directed to the corresponding authors.

## Author contributions

LG: Conceptualization, Data curation, Investigation, Methodology, Writing – original draft. JH: Data curation, Investigation, Methodology, Software, Writing – original draft. YW: Data curation, Investigation, Writing – review & editing. YC: Methodology, Software, Writing – review & editing. WQ: Formal analysis, Writing – review & editing. CM: Software, Supervision, Writing – review & editing. PF: Methodology, Supervision, Writing – review & editing. YJ: Investigation, Supervision, Writing – review & editing.
